# When culture meets child rights: Confucian ethics and legal challenges in mental health protection for minors in China

**DOI:** 10.3389/fpubh.2025.1691211

**Published:** 2025-10-20

**Authors:** Taoying Li, Long Zheng

**Affiliations:** ^1^Law School, Xinjiang University, Urumqi, Xinjiang, China; ^2^Department of Law, Jiangxi Police Institute, Nanchang, Jiangxi, China; ^3^Nanchang Institute of Science and Technology, Nanchang, Jiangxi, China

**Keywords:** Confucian values, minors’ rights, mental health, cultural adaptation, China

## Abstract

Against the backdrop of growing global awareness of children’s rights protection, mental health issues among minors in China have gradually gained social attention. However, the current legal system faces significant tensions between institutional provisions and cultural adaptations, resulting in the poor enforcement of certain legal provisions and a failure to effectively address the psychological challenges faced by adolescents. This paper explores the dual impact of Confucian values on adolescents’ mental health: on one hand, its emphasis on respecting teachers, valuing education, diligence in learning, and adherence to etiquette helps maintain educational order; on the other hand, its hierarchical obedience and shame-oriented approach may suppress individual emotional expression and hinder psychological support behaviors. Based on an analysis of the root causes of the conflict between China’s current legal system and cultural norms, this paper adopts an interdisciplinary perspective, integrating developmental psychology, children’s rights law, and Confucian ethics to propose a “culturally adaptive mental health rights protection framework.” This framework aims to achieve effective protection of minors’ mental health while facilitating the modern transformation of traditional culture through a combination of legal empowerment and cultural change. This paper proposes a culturally adaptive framework for safeguarding minors’ mental health rights, clarifying how tensions between Confucian ethics and the Convention on the Rights of the Child (CRC) can be reconciled through layered approaches at both the institutional and practical levels. It aims to provide theoretical grounding and policy guidance for the localized construction of China’s child-rights protection mechanisms.

## Introduction

In recent years, mental health issues among Chinese primary and secondary school students have become increasingly prominent, posing a significant public health challenge (1). According to the 2021–2022 China National Mental Health Development Report, approximately 14.8% of minors exhibit depressive symptoms. Globally, the average prevalence rate of non-suicidal self-injury among minors is 19.5%, whereas the prevalence rate among Chinese middle school students (aged 13 to 18) reaches as high as 27.4% (2), highlighting a more severe situation. Research indicates that academic pressure and school bullying are the two primary risk factors contributing to mental health issues among minors (3). In response to this trend, the national government has continuously introduced intervention measures. The General Office of the State Council and the General Office of the Ministry of Education have successively issued the Opinions on Further Reducing the Burden of Homework and Extracurricular Training for Students in the Compulsory Education Stage and the Notice on Strengthening the Management of Students’ Mental Health, both aimed at alleviating external pressures and improving the mental health support system.

Meanwhile, the newly revised Minor Protection Law of 2020 systematically stipulates and institutionally safeguards the mental health rights of minors from five dimensions: family, government, school, society, and the judiciary (4). However, these system improvements have not fundamentally resolved the deep-seated cultural conflicts. In actual educational practice, traditional Confucian values continue to profoundly influence family upbringing methods, school educational philosophies, and societal standards for what constitutes a “good child.” These values, on one hand, emphasize family cohesion, moral education, and social harmony, which help create a stable environment for growth, provide emotional support, and offer value recognition for adolescents, thereby contributing to enhancing their psychological resilience (5).

On the other hand, certain core norms within Confucian culture, in certain circumstances, restrict adolescents’ self-expression, suppress their emotional needs, and even weaken their willingness to seek psychological support (6). These factors exert complex and enduring effects on mental health (7).

For a long time, Confucian educational concepts have been deeply rooted in China’s educational system, leading education to prioritize “discipline maintenance” and “behavioral norms,” while relatively neglecting “individual rights” and “mental health.” (8) In this educational value system, collectivism and moral education remain dominant (9).

When China ratified the United Nations Convention on the Rights of the Child, it emphasized the “principle of the best interests of the child” but made reservations regarding Article 14, which advocates respecting children’s rights to freedom of thought, expression, and participation (i.e., children’s right to freedom of thought). This reservation reflects the clash between traditional cultural values and modern concepts of rights. It underscores the urgent institutional challenge in contemporary China: how to effectively implement the mental health rights of minors while respecting traditional culture.

Theoretically, existing research largely remains at the level of institutional provision in addressing minors’ mental-health rights, lacking a systematic account of the cultural conditions and value-negotiation mechanisms essential to realizing these rights. Practically, advances in legal texts have not been matched by concurrent transformation of interactional norms in family–school settings, producing a disconnect between institutional expectations for expression and participation rights and the everyday structures of educational culture. Accordingly, constructing a culturally adaptive framework for rights realization can both fill theoretical gaps and provide an operational bridge for policy implementation. This paper systematically reviews the current state of Chinese youths’ mental health and the influence of traditional Confucian culture, analyzes the institutional shortcomings of existing laws on the protection of minors in addressing deep-seated cultural factors, and explores how to integrate legal studies, developmental psychology, and Confucian ethics to tackle practical challenges in legal implementation. The goal is to offer culturally adaptive and practically targeted theoretical foundations and policy recommendations for building legal safeguards for minors’ mental-health rights in China, thereby mitigating adverse impacts on their mental well-being.

## The impact of Confucian values on the mental health of minors in China

Confucianism, as the most representative ethical system in traditional Chinese culture, has profoundly shaped China’s family structure, educational philosophy, and patterns of interpersonal relationships. Its core concept emphasizes that society is a hierarchical, interdependent network of relationships. This principle is not only reflected in parent–child relationships but also extends to respect and obedience toward teachers, elders, and all forms of authority (6). This cultural norm constructs a “self-in-relation” identity—individuals are understood as members embedded within family and collective networks, with their value and role deriving from their relationships with others, rather than from isolated individual autonomy.

Although this relational self-concept helps enhance individuals’ sense of social belonging and responsibility, it also exerts a profound dual impact on the psychological development of adolescents.

On the one hand, Confucian values, such as respecting teachers, honoring the older adults, being filial, and diligent study, have long provided positive cultural resources for youth education (10). These values encourage students to obey discipline, respect authority, and regard academic achievement as an important manifestation of family honor (11). Under this cultural influence, primary and secondary school students generally exhibit a high degree of obedience and diligence (12). Many children are taught from a young age to follow the guidance of their parents and teachers, viewing “studying hard” as both a family obligation and an expression of filial piety (13). Confucian ideals, such as “hoping one’s son will become a dragon,” remain deeply ingrained in family education practices, motivating students to invest significant time and effort in pursuing academic success (14). In school settings, this collectivist orientation reinforces students’ awareness of teamwork, emphasizing the importance of class honor and group harmony, thereby reducing conflict and tendencies toward individual deviant behavior (12). Confucian thought also emphasizes the mutual responsibilities of parental kindness (fatherly righteousness and motherly kindness) and filial piety, prompting Chinese parents to invest substantial resources in their children’s education and well-being. For adolescents, this translates into a strong sense of value and security (15). Additionally, it encourages viewing adversity as an opportunity for self-cultivation. This perceiving setbacks as tests of virtue rather than as disasters—and fosters a sense of life meaning that transcends the self (16).

**Figure 1:**
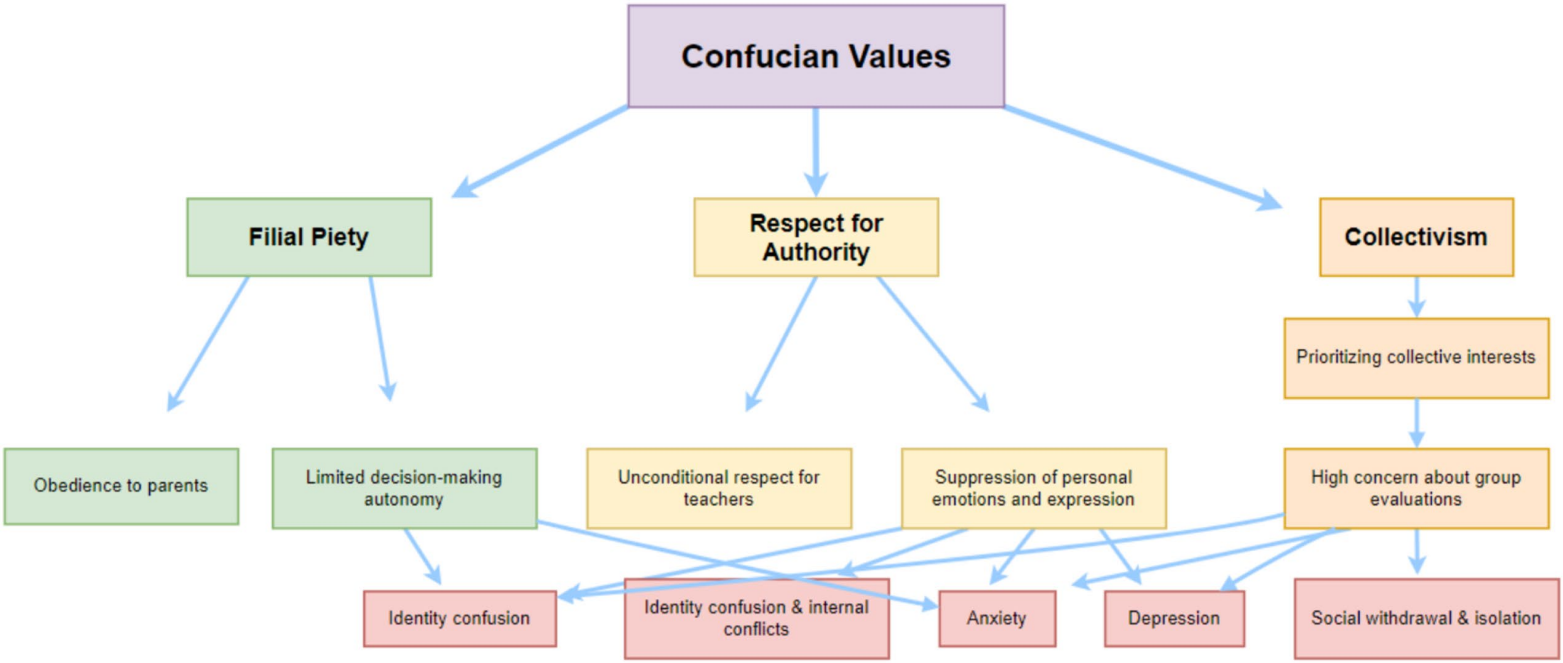
The impact of Confucian values on the physical and mental health of minors.

However, while Confucian culture provides important support, it can also inhibit psychological expression and effective intervention when certain norms are overemphasized or misused. (1) Confucian thought places great emphasis on “harmony” and social order, fostering a cultural preference for emotional restraint. From a young age, Chinese adolescents are taught to “endure” and “not cause trouble for the family,” leading many to adopt emotional suppression strategies: habitually hiding or downplaying pain, disappointment, or dissent (17). Long-term emotional suppression is associated with increased internalized symptoms because individuals fail to effectively process or release emotions (18). (2) The hierarchical structure and authority-oriented nature of Confucian culture can hinder the development of autonomy in extreme cases. Many Chinese parents, out of love and responsibility, overly intervene in their children’s lives. This excessive obedience to authority, over-accommodation of parental expectations, suppression of personal emotions, and blind conformity to collective norms may lead adolescents to neglect their own feelings (11, 19), lack independent judgment and emotional regulation skills, and experience identity confusion during their development (20). (3) One of the most harmful cultural barriers is the stigma associated with mental illness. In Confucian society, personal value is closely tied to fulfilling social roles, and mental health issues are viewed as failures of personal and family education. This stigma manifests in various ways: families may deny their children’s mental health issues to avoid “losing face”; adolescents may view psychological distress as a sign of weakness and be reluctant to discuss it (21). This tendency is particularly evident in the increasingly severe mental health issues among adolescents today (Figure 1).

## The United Nations convention on the rights of the child—minors’ right to participation and expression

Modern theories of children’s rights, as represented by the United Nations Convention on the Rights of the Child, assert that children should not merely be passive recipients of protection, but rather independent individuals with the right to participate, speak out, and express themselves on matters that concern them (22).

China ratified the Convention in 1992 and has gradually incorporated its principles into its domestic legal system. For example, Article 3 of the revised Minor Protection Law of 2020 explicitly stipulates that minors enjoy the rights to survival, development, protection, and participation. This marks the first time that Chinese law has formally recognized children’s right to participation. In a society long influenced by Confucian patriarchy, this legislative change holds far-reaching significance.

From a children’s rights perspective, adolescents should be granted progressive autonomy: they should be allowed and encouraged to express their feelings (including psychological distress), participate in life decisions (such as educational or medical choices), and freely seek help, with confidentiality and without discrimination. This rights-based participation can improve mental health by empowering adolescents, recognizing the value of their experiences, and promoting early intervention. However, in practice, there is often a disconnect between law and culture. Deep-rooted cultural norms do not necessarily change immediately with legal revisions. In many Chinese families and schools, adult authority continues to overshadow children’s participation—children “voicing their opinions” may be seen as inappropriate or insignificant. The principles of child autonomy and expression advocated by the Convention on the Rights of the Child conflict with Confucian cultural norms that emphasize obedience and collective priority. This conflict means that, despite legal progress, adolescents may still hesitate to discuss mental health issues, and adults may underestimate the importance of listening to adolescents’ voices (23). Cross-national research indicates that the local implementation of children’s rights must adapt to prevailing ethical frameworks; its effectiveness hinges on the mutual accommodation of institutional norms and local culture (24–27). This underscores the need for a prudent strategy to reconcile children’s rights with Confucian family values.

## The value conflict between China’s legal system and cultural inertia

This paper defines “value conflict” as the systematic clash between two normative orders—such as Confucian ethics and the Convention on the Rights of the Child’s rights framework—across their value objectives, bases of legitimacy, and behavioral expectations. In recent years, China has made rapid progress in legal reforms aimed at protecting the rights and interests of adolescents. The revised Minor Protection Law (2020) established the principles of child protection and participation, explicitly requiring the safeguarding of minors’ physical and mental health and the consideration of children’s opinions in relevant decision-making processes. The “quality education” reform (the “double reduction” policy of 2021) was implemented to strengthen measures aimed at alleviating student stress. The Mental Health Law (implemented in 2013) safeguards citizens’ (including adolescents’) right to access mental health services, requires schools and communities to promote mental health, and emphasizes the principles of informed consent and minimal restriction for patients with mental disorders. Additionally, the implementation of specialized plans, such as the Action Plan for the Mental Health of Children and Adolescents (2019–2022), has promoted the development of school counseling services, the training of professional personnel, and the enhancement of mental health awareness. Although a relatively comprehensive legal and policy framework has been established to safeguard adolescents’ mental health, implementing these modern concepts within the context of traditional culture still faces numerous challenges.

**Figure 2:**
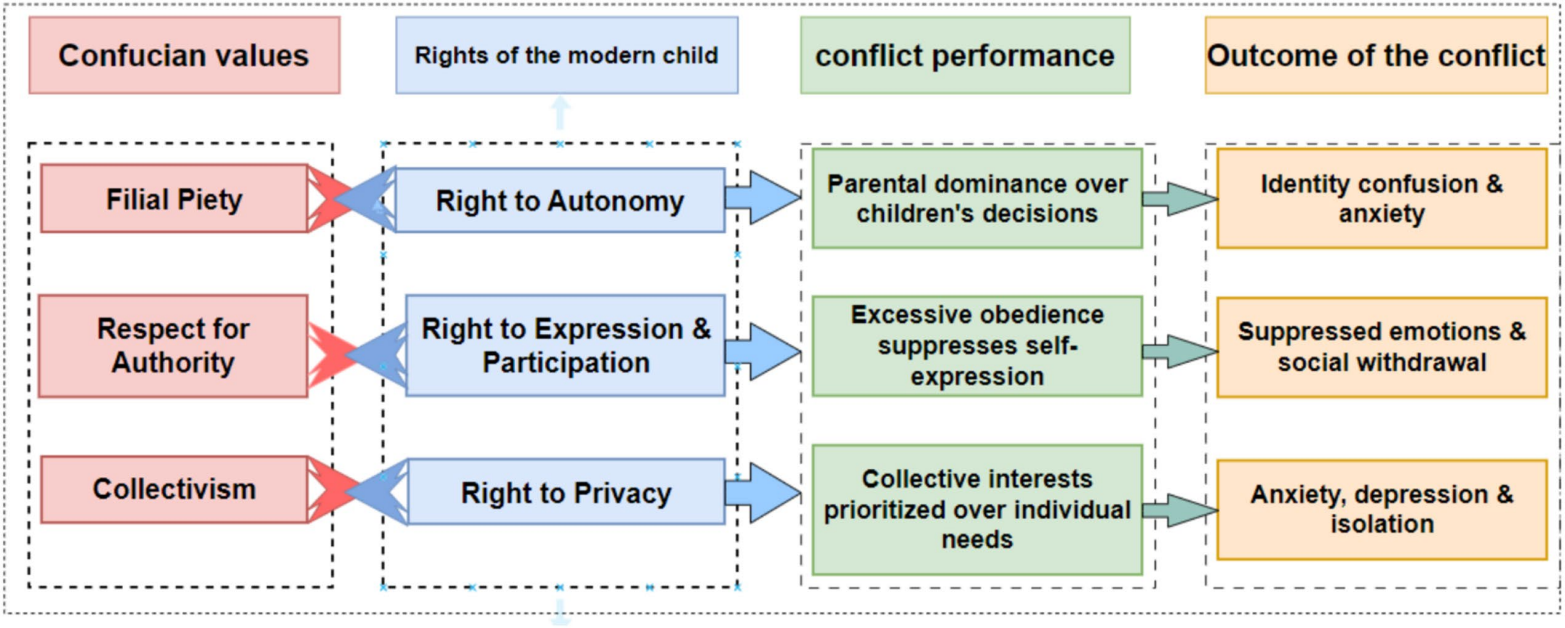
Conflict between Confucian values and modern children’s rights.

1. Challenges in Implementing the Right to Participation. Although the law recognizes children’s right to participation and the concept of “child participation” has entered the policy discourse, Confucian family ethics place parental authority at the core and traditionally do not recognize minors as having independent will or basic rights claims (28). In reality, schools still widely adopt a top-down management model (29). The deeply ingrained notion of “elder authority” often supersedes legal requirements, and parents continue to make decisions on behalf of their children in important matters such as course selection and mental health interventions.
2. Resistance to the Transformation of Educational Philosophy. Despite the explicit prohibition of corporal punishment and the promotion of social–emotional learning, some educators continue to insist that strict discipline (including corporal punishment) is necessary and effective (30). Meanwhile, although schools generally have counseling rooms, their actual usage rates remain limited due to insufficient cultural acceptance. Surveys indicate that students with psychological issues often fail to receive timely assistance, which is attributed to a lack of awareness and the stigma associated with mental health issues (31), thereby exacerbating the severity of these problems (32).
3. Structural Contradictions in Academic Pressure. Despite policies advocating holistic development, the “education fever” fueled by the Confucian examination system tradition and contemporary social competition continues to drive families to prioritize academic performance (13). This cultural value system, which views educational achievements as a manifestation of filial piety, conflicts with mental health awareness, creating a paradox where policy-driven stress reduction collides with societal pressure.
4. Dilemmas in Privacy Rights Practices. Modern concepts emphasize that minors have the right to privacy and personal space, but under Confucian culture, family boundaries are relatively blurred. Traditional Chinese family values adhere to a privacy philosophy that emphasizes both collectivism and parental authority: family members are expected to be open and honest with each other and mutually dependent, and children’s affairs are typically regarded as the affairs of the entire family (33). As a result, the violation of minors’ privacy has become the norm, and the obscuring effect of family collectivism on individual rights remains significant (Figure 2).

## Discussion: building a culturally adaptive mental health support system for adolescents

1. Implementation Conditions and Potential Barriers. Weak professional mental-health resources at the county and township levels, coupled with inadequate referral networks, disrupt the continuum of early identification and intervention. School evaluation systems prioritize academic performance, squeezing teachers’ time and rendering students’ rights to express concerns and seek help mere formalities, thereby marginalizing psychological services. A high-power-distance culture and a “harmony-first” mindset suppress emotional expression and proactive help-seeking, often concealing risk indicators that go undetected by peers and teachers. Furthermore, standardized procedures and documentation for home–school communication are lacking, blurring boundaries between privacy and information sharing. Unclear departmental responsibilities increase the risks of data misuse and algorithmic bias. The absence of evaluation metrics that balance “rights fulfillment” with “cultural acceptability” may produce compliance gaps and distorted performance incentives.
2. Culturally Embedded Legal and Educational System Design. In the field of mental health protection for minors, this means that formal systems should not only be based on universal human rights standards but also be deeply embedded in local value systems. When implementing Article 12 of the Convention on the Rights of the Child regarding the right to expression, mechanisms such as “senior peer support groups” and student-participatory psychological feedback systems can be employed to ensure that the practice of the right to expression does not conflict with traditional ethical norms of respecting teachers and elders, thereby achieving a balance between legal norms and cultural identity. Additionally, “caring for children’s mental health” can be incorporated into the evaluation criteria for “civilized families,” parental education modules can be integrated into psychological interventions, and community authority can be leveraged to reduce the “stigma associated with mental illness,” thereby promoting the transformation of traditional filial piety into modern family responsibility. Furthermore, an elective course titled “Traditional Culture and Children’s Psychological Development” could be established in teacher training institutions, using case-based teaching to explain the “benevolent yet not strict” approach to classroom management. Such institutional adaptations can facilitate the advancement of legal objectives while respecting cultural traditions.
3. Establishing a Cultural Mediation Mechanism Centered on “Community Confucian Scholars.” We can draw on traditional mechanisms such as “village agreements” and “clan admonitions” to introduce a “community Confucian scholar mediation mechanism” as a form of soft governance intermediary in the field of child protection in cases of domestic violence, school bullying, or psychological alienation. Specifically, “cultural mediation groups” could be established at the street or township level, composed of retired teachers, respected community elders, and child welfare supervisors, to conduct counseling and psychological interviews for cases of psychological abuse or family cold violence identified at an early stage. Additionally, the “Community Family Psychological Guidelines,” based on the principles of “propriety, righteousness, benevolence, and harmony,” could be introduced to guide families in addressing children’s emotions and rebellious behavior through non-violent means. Before severe psychological harm cases enter the judicial process, community mediators could issue cultural intervention reports to provide foundational support for family re-education and behavioral change, thereby enhancing the cultural legitimacy of institutional interventions.
4. Mechanism for Coordinating Traditional Culture and International Norms. China can proactively present its cultural adaptation path in its report on the implementation of the United Nations Convention on the Rights of the Child, particularly in the implementation of key provisions such as Article 3 (best interests of the child), Article 12 (right to expression), and Article 24 (right to health), embedding practical models with Chinese cultural characteristics. In the revision project of the Children’s Code by the Legal Affairs Committee of the National People’s Congress, it is essential to systematically review clause language compatible with Confucian concepts such as “benevolence,” “self-cultivation,” and “understanding reason” to establish a cultural channel for the style of legal provisions. Additionally, the implementation of “localization of children’s issues legal experiment projects” should be promoted.

## Conclusion

The realization of minors’ rights is not a single, universal process but rather one that is culturally contextualized. In this sense, Confucian ethics are not merely obstacles to modern minors’ rights but rather cultural factors that must be acknowledged and understood in the process of realizing these rights. Therefore, this study advocates the construction of a more inclusive and flexible “culturally adaptive” paradigm, which establishes a dynamic balance between international standards and local traditions, ensuring that the realization of minors’ rights complies with international norms while remaining compatible with local cultural ethics. Of course, the “cultural adaptation” invoked here does not entail compromising core values but rather making limited adjustments premised on non-negotiable rights boundaries. In non-negotiable domains—corporal punishment, forced treatment, and systemic privacy violations—a zero-tolerance stance must be maintained. Only within rights-compliant space should friction be reduced through procedural and role-based arrangements, accompanied by phased evaluations to assess whether such flexibility is warranted and effective.

## Limitations of the study

This study has certain limitations that should be addressed in future research.

First, the data and literature analyzed to date are predominantly macro-level and general in nature, with no comparative analysis conducted between economically developed regions and rural areas. Future research should focus on quantitative analysis and detailed comparisons of regional differences.

Second, this study has not yet conducted a quantitative analysis of behavioral differences in the implementation of minors’ rights and mental health issues across regions with varying degrees of Confucian cultural influence. Future research should employ quantitative methods and cross-regional comparative studies to systematically explore the relationship between the extent of Confucian cultural influence and the severity of mental health issues among minors.

Third, this study primarily focuses on theoretical analysis and case illustrations, lacking large-scale data support and long-term tracking research. Future research should further emphasize empirical studies and longitudinal investigations to more comprehensively understand the dynamic relationship between culture, law, and mental health.

Finally, this paper primarily examines adolescents’ mental health and rights protection and does not systematically explore how educational culture interacts with rights at the preschool and elementary stages. Future research could employ longitudinal cohort designs to trace the causal chain linking early parenting styles → expression strategies → help-seeking behaviors → intervention outcomes.

## Data Availability

The original contributions presented in the study are included in the article/supplementary material, further inquiries can be directed to the corresponding author.
